# From simulator to patient: transfer of procedural skills to practice in cardiac device implantation

**DOI:** 10.1093/ehjopen/oeag120

**Published:** 2026-07-29

**Authors:** Jorio Mascheroni, Martin Stockburger, Ashish Patwala, Hartwig Retzlaff, Anthony G Gallagher

**Affiliations:** Faculty of Medicine, KU Leuven, Herestraat 49, Leuven 3000, Belgium; Department of Cardiac Rhythm Management Training & Education, Medtronic International Trading Sàrl, Route du Molliau 31, Tolochenaz 1131, Switzerland; Department of Cardiology and Internal Medicine, Havelland Kliniken, Ketziner Straße 21, Nauen 14641, Germany; Institute of Medical Sociology and Rehabilitation Science, Charité – Universitaetsmedizin Berlin, Charitéplatz 1, Berlin 10117, Germany; Department of Cardiology, University Hospital of North Midlands, Newcastle Road, Stoke-on-Trent ST4 6QG, UK; Training Concept Consulting, Alpenstrasse 14a, Groebenzell D-82194, Germany; Faculty of Medicine, KU Leuven, Herestraat 49, Leuven 3000, Belgium; School of Medicine, Faculty of Life and Health Sciences, Ulster University, Magee Campus, Northland Rd, Londonderry BT48 7JL, UK

**Keywords:** Pacing, Cardiac resynchronization therapy, Training, Skill, Proficiency-based progression, Metrics

## Abstract

**Aims:**

Cardiac implantable electronic devices (CIED) training for novice implanters typically occurs *in vivo*, varies across countries/institutions, and results in inconsistent skill levels. Proficiency-based progression (PBP) simulation training—requiring attainment of expert-derived performance benchmarks before clinical practice—reduced procedural errors by 61–77% compared with traditional simulation-based training (SBT) in randomized simulation trials. This study aimed to quantify clinical skill transfer in CIED implantation for the first time, comparing *in vivo* deviations from expert-agreed optimal technique in early-career implanters previously randomized to PBP vs. SBT.

**Methods and results:**

Prospective observational cohort study following participants from a prior multinational randomized simulation trial to assess transfer of training to clinical practice. Early-career implanters (≥20 prior pacemaker/defibrillator cases; limited CRT first-operator experience) reported the implant technique they applied in their first two routine clinical cases within 30 days of training, using a validated standardized performance metrics form assessing surgical and lead implantation steps. The primary outcome was the mean percentage of deviations from optimal technique per participant.

Of 30 randomized participants, 21 (70%) submitted 42 case reports (10 PBP, 11 SBT). Baseline characteristics were similar, and case complexity did not differ between groups (*P* = 0.43). PBP trainees demonstrated 60% fewer procedural deviations than SBT trainees (mean [SD], 5.8% [3.8%] vs. 14.4% [9.1%]; *P* = 0.013).

**Conclusion:**

Metrics-based PBP training was associated with significantly fewer procedural deviations from optimal CIED implant technique during early clinical practice compared with traditional simulation. These findings support benchmark-based simulation as a strategy to improve early clinical skill transfer in cardiac electrophysiology.

## Graphical Abstract

**Graphical Abstract:**
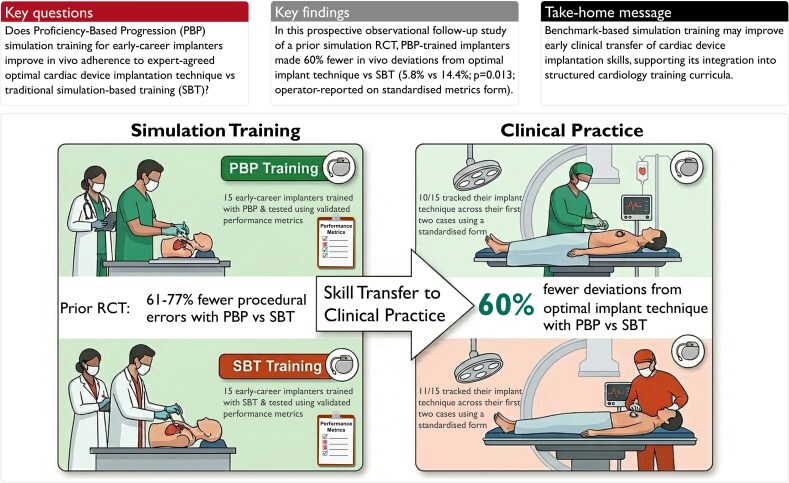

## Introduction

Cardiac implantable electronic devices (CIEDs) are established therapies for selected patients with cardiac rhythm disorders, but complications related to surgical and implant technique remain an important clinical burden, particularly early in the operator learning curve. Technical skill is clinically relevant because operator performance has been associated with patient outcomes,^1^ and competence assessments have shown substantial deficits in early-career device implanters.^2^

Proficiency-based progression (PBP) simulation training was developed to address this gap by requiring trainees to achieve an expert-derived benchmark on validated procedural metrics (steps, errors, critical errors) before advancing to patient care.^3,4^ Across procedural medicine, PBP has consistently improved technical proficiency compared with conventional simulation training.^5^ Unlike traditional time- or volume-based apprenticeship models, PBP links progression to objectively demonstrated performance—an approach recently advocated by the European Heart Rhythm Association (EHRA) as the framework for standardized electrophysiology and CIED training.^6^ In randomized simulation studies of CIED implantation, PBP reduced lead implantation and surgical errors compared with traditional simulation by 77% and 61%, respectively.^7,8^ Whether these simulation gains translate to clinical practice has not previously been evaluated in CIED implantation. We therefore sought to assess *in vivo* procedural deviations from expert-agreed optimal technique^9,10^ in early-career operators previously trained with PBP vs. traditional simulation-based training (SBT).

## Methods

### Study design and participants

This prospective observational cohort study constituted the final component (clinical skill transfer) of a CIED implantation training curriculum, supplemental to usual institutional training, and was conducted between April and December 2022. Data were analysed between January and March 2023. Participants were early-career implanters who had been randomized in a prior multinational simulation-based trial to one of two training pathways: PBP or SBT.^7^ Both groups had completed e-learning and simulation training components based on the same content and agenda, but progression criteria differed. In the PBP pathway, trainees advanced only after achieving a prespecified quantitative proficiency benchmark, verified by instructors using validated, objective performance metrics.^10^ In the SBT pathway, progression occurred at proctor discretion without consistent, objective performance targets. The trial had ethics approval from KU Leuven, Belgium (ClinicalTrials.gov: NCT05952908). All participants provided written informed consent for the full training curriculum. This study is reported in accordance with the STROBE guidelines. Baseline characteristics of the 30 participants, representing 10 countries, have been reported previously.^7^ Briefly, the cohort comprised early-career implanters with prior first-operator experience in pacemaker/defibrillator procedures but limited prior first-operator experience in cardiac resynchronization therapy (CRT).

### Data collection

**Figure 1:**
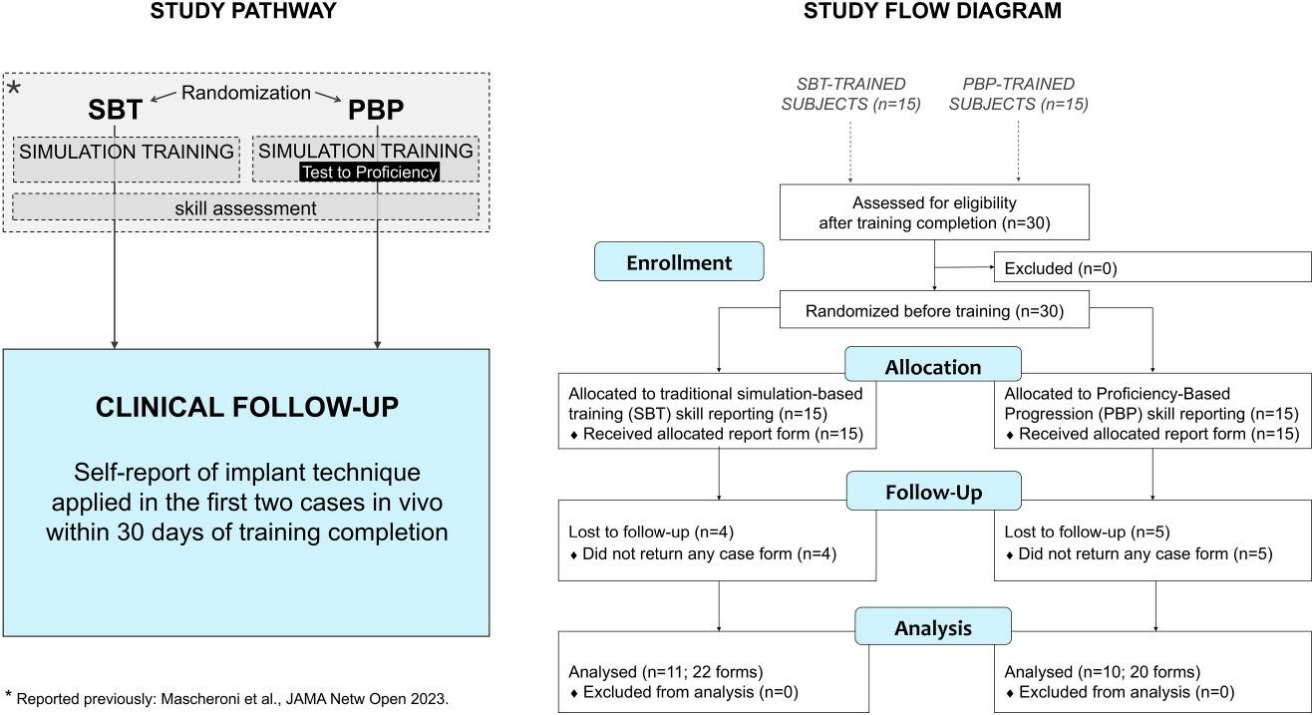
Study pathway and study flow diagram. Abbreviations: PBP, proficiency-based progression simulation training group; SBT, traditional simulation-based training group.

Participants were asked to self-report the implant technique used in their first two routine clinical cases within 30 days of simulation training completion to minimize skill decay, preferably CRT procedures and otherwise standard pacemaker or defibrillator implants. To encourage veridical reporting, submissions were confidential, carried no pass-fail consequences, and were analysed only in aggregate and anonymously. At follow-up, participants remained unaware of their original randomized training assignment. The study did not interfere with routine clinical care, and no patient or procedure-specific identifiers were collected. *Figure 1* illustrates the study pathway and participant flow.

Performance was captured using a standardized performance metrics form covering the implant technique used throughout the procedure, derived directly from validated PBP metrics and structured to reduce recall bias. Rather than relying on unrestricted free recall, participants selected from predefined procedural options associated with expert-agreed performance metrics.^9,10^ The form covered five sections: general workflow, surgical steps, and right ventricular (RV), right atrial (RA), and left ventricular (LV) lead placement. It included up to 23 checkpoints for a three-lead implantation.

A deviation was defined as any step performed differently from the expert-agreed optimal technique, including procedural choices such as venous access approach, suturing method, or lead fixation technique. Deviations were used as a proxy for adherence to optimal technique and were not intended to be interpreted as clinical complications.

### Outcomes

The primary outcome was percentage deviation rate, calculated per case as deviations divided by total applicable steps and averaged per participant into a single composite score.

### Statistical analysis

Groups were compared using Welch *t*-tests because of unequal variances, and device-type distributions were compared using *χ*^2^ testing. Two-sided *P* < 0.05 was considered statistically significant. Data were analysed using JASP (version 0.19.4). Additional *post hoc* analyses were performed using independent-samples *t*-tests to compare procedure and fluoroscopic duration between groups within implant type and overall, and correlation analysis to examine the association between years of device practice and deviations from optimal technique.

## Results

### Participants

Of 30 trainees in the randomized trial, 21 submitted 42 clinical case reports within 30 days of training completion (10/15 PBP [67%], 11/15 SBT [73%]). Nine participants did not submit cases within this timeframe and were excluded from the analysis. Baseline characteristics were similar between groups and were reported previously.^7^ Years of experience in device practice did not differ significantly between trainees who did and did not submit reports.

### Case mix and complexity

Reported cases comprised 29 CRT systems (22 three-lead, 6 two-lead, and 1 upgrade), 11 dual-chamber devices, and 2 single-chamber devices. Case complexity did not differ between groups (*χ*^2^ test, *P* = 0.43).

### Study outcomes: *in vivo* procedural deviations

**Figure 2:**
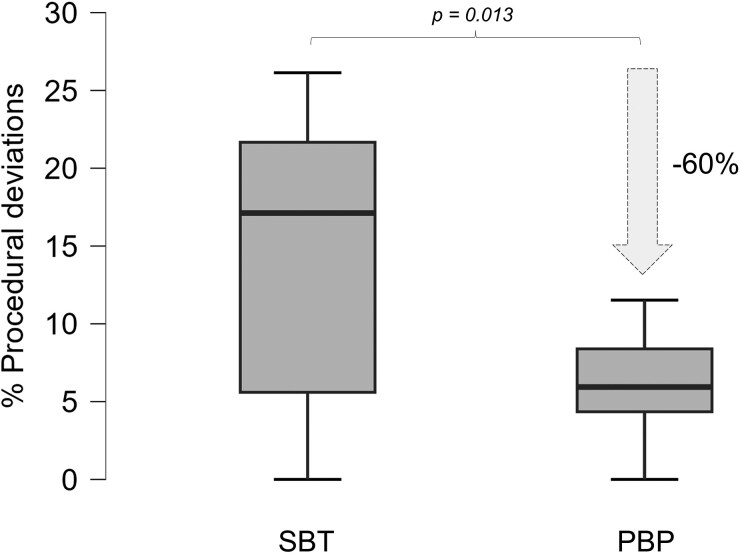
Primary study outcome: percentage of deviations from expert-agreed optimal implant technique by training group. Box plots indicated medians (central line) and 75% and 25% quartiles (upper and lower ends); whiskers represent maximum and minimum values. Abbreviations: PBP, proficiency-based progression simulation training group; SBT, traditional simulation-based training group.

For the primary outcome, mean [SD] deviation rates were 5.8% [3.8%] in the PBP group vs. 14.4% [9.1%] in the SBT group, corresponding to a 60% lower deviation rate with PBP (Welch *t* = −2.839, df = 13.71, *P* = 0.013; Cohen's *d* = 1.22), as shown in *Figure 2*. *Table 1* lists the types of deviations and the number of occurrences observed in each group.

**Table 1:** Types and numbers of deviations from optimal technique in each group

Deviation	SBT (*n* = 22)	PBP (*n* = 20)
Injected local anaesthesia using >3 punctures	1	0
Selected subclavian vein for venous access	11	9
Did not tie separate knots to anchoring sleeve & muscle during lead fixation	3	0
Placed device submuscular without clinical reason	0	1
Used monofilament suture to close subcutaneous tissues	4	2
Implanted LV lead before RV lead	1	1
Did not verify final RV lead position in LAO fluoro view	10	3
Did not monitor RV lead markers under fluoroscopy during helix deployment	2	0
Implanted RA lead on lateral wall without clinical reason	1	0
Did not monitor RA lead markers under fluoroscopy during helix deployment	3	0
P-wave amplitude was below minimum recommended	4	0
Coronary sinus cannulated using guide catheter only (no softer tool inside)	1	0
Did not confirm coronary sinus catheter access using contrast or distal wire	2	0
Did not perform occlusive coronary sinus venogram	3	4
Did not perform coronary sinus venogram in two fluoroscopy views	2	0
LV lead implanted in anterior or posterior vein	1	0
Did not confirm final LV lead position in two fluoroscopy views	2	0
Phrenic nerve stimulation threshold <8 V at LV lead site	3	2
Needed hands-on intervention by supervisor during the procedure	7	2

Abbreviations: LV, left ventricular; PBP, proficiency-based progression simulation training group; RA, right atrial; RV, right ventricular; SBT, traditional simulation-based training group.

*Post hoc* analysis showed no significant association between years of device practice and the number of deviations from optimal technique, and no significant differences in procedure or fluoroscopic duration within each implant type or overall.

## Discussion

In this prospective follow-up of early-career CIED implanters, PBP simulation training was associated with superior early clinical transfer compared with traditional simulation. Operators trained with PBP to an expert-derived performance benchmark made 60% fewer procedural deviations from optimal technique (5.8% vs. 14.4%) than SBT-trained peers during their first routine clinical cases. This lower deviation rate with PBP vs. SBT is consistent with our prior simulation-based trial findings (77% and 61% error reductions)^7,8^ and with the 60% reduction in performance errors reported in a meta-analysis of randomized PBP studies.^5^

A plausible explanation for this difference is the objective metrics-driven coaching and benchmark-based progression inherent to PBP, which underpinned better clinical skills. In traditional simulation, trainees may advance after a fixed duration or number of cases regardless of the performance level achieved. Under PBP, progression requires demonstrated proficiency. This approach may produce more durable motor and cognitive patterns that remain resistant to decay and interference when operators encounter the variability and complexity of live cases, including anatomic variation, time pressure, and uneven supervision.

These findings also support the broader principle of transfer of training previously demonstrated in surgery and anaesthesia^3,5^ and suggest that benchmark-based simulation may improve early adherence to optimal implant technique in electrophysiology. The observed errors were not trivial stylistic differences; they involved procedural decisions with potential implications for implant quality and, plausibly, downstream complications. Although this study was not designed to evaluate clinical events, the findings support the concept that better procedural preparation and skill before patient exposure may improve consistency and quality of early operator performance.

Future prospective studies should evaluate whether deviations measured in this way correlate with independently assessed performance and patient complications.

### Limitations

Performance was self-reported rather than independently observed, although the multiple-choice structure and confidentiality were intended to reduce recall and social desirability bias. In addition, 30% of randomized participants did not submit follow-up cases, although attrition was balanced between groups. The *in vivo* follow-up was observational and should be interpreted accordingly.

## Conclusions

Skills acquired through PBP simulation training transferred effectively to early clinical CIED implantation. Compared with traditional simulation, PBP was associated with significantly fewer *in vivo* deviations from optimal technique, supporting benchmark-based simulation as a strategy to standardize procedural performance with potential implications for patient safety.

## Lead author biography


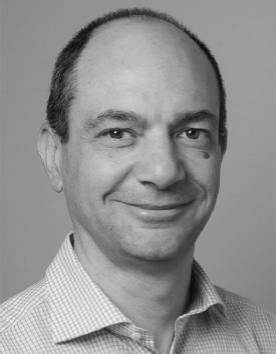


Dr Jorio Mascheroni holds an MSc in Biomedical Engineering from Politecnico di Milano (Italy), an MSc in Technology Enhanced Learning for Health from University College Cork (Ireland), and a PhD in Biomedical Sciences from KU Leuven (Belgium). His doctoral research focused on the development and validation of a proficiency-based progression simulation training curriculum for novice cardiac device implanters. Passionate about training and education, he has spent more than 20 years in the medical industry collaborating with experienced clinicians to train healthcare professionals in cardiac device therapies. He currently works in the Cardiac Rhythm Management department of Training & Education at Medtronic International, Switzerland.

## Data Availability

The data that support the findings of this study are available from the corresponding author upon reasonable request.
